# Deep learning-enhanced radiomics for histologic classification and grade stratification of stage IA lung adenocarcinoma: a multicenter study

**DOI:** 10.3389/fonc.2023.1224455

**Published:** 2023-07-20

**Authors:** Guotian Pei, Dawei Wang, Kunkun Sun, Yingshun Yang, Wen Tang, Yanfeng Sun, Siyuan Yin, Qiang Liu, Shuai Wang, Yuqing Huang

**Affiliations:** ^1^ Department of Thoracic Surgery, Beijing Haidian Hospital (Haidian Section of Peking University Third Hospital), Beijing, China; ^2^ Institute of Advanced Research, Infervision Medical Technology Co. Ltd., Beijing, China; ^3^ Department of Pathology, Peking University People’s Hospital, Beijing, China

**Keywords:** deep learning, artificial intelligence, radiomics, model, lung adenocarcinoma

## Abstract

**Background:**

Preoperative prediction models for histologic subtype and grade of stage IA lung adenocarcinoma (LUAD) according to the update of the WHO Classification of Tumors of the Lung in 2021 and the 2020 new grade system are yet to be explored. We aim to develop the noninvasive pathology and grade evaluation approach for patients with stage IA LUAD via CT-based radiomics approach and evaluate their performance in clinical practice.

**Methods:**

Chest CT scans were retrospectively collected from patients who were diagnosed with stage IA LUAD and underwent complete resection at two hospitals. A deep learning segmentation algorithm was first applied to assist lesion delineation. Expansion strategies such as bounding-box annotations were further applied. Radiomics features were then extracted and selected followed by radiomics modeling based on four classic machine learning algorithms for histologic subtype classification and grade stratification. The area under the receiver operating characteristic curve (AUC) was used to evaluate model performance.

**Results:**

The study included 294 and 145 patients with stage IA LUAD from two hospitals for radiomics analysis, respectively. For classification of four histological subtypes, multilayer perceptron (MLP) algorithm presented no annotation strategy preference and achieved the average AUC of 0.855, 0.922, and 0.720 on internal, independent, and external test sets with 1-pixel expansion annotation. Bounding-box annotation strategy also enabled MLP an acceptable and stable accuracy among test sets. Meanwhile, logistic regression was selected for grade stratification and achieved the average AUC of 0.928, 0.837, and 0.748 on internal, independent, and external test sets with optimal annotation strategies.

**Conclusions:**

DL-enhanced radiomics models had great potential to predict the fine histological subtypes and grades of early-stage LUADs based on CT images, which might serve as a promising noninvasive approach for the diagnosis and management of early LUADs.

## Introduction

Lung cancer remained the leading cause of cancer death worldwide with annually 2.1 million new lung cancer cases and 1.8 million deaths (1). Unfortunately, approximately 70% of these patients are diagnosed with locally advanced stages and metastatic disease, which results in low survival rates (2). Thus, early detection and treatment of lung cancer are essential to reduce mortality. With the widespread development of low-dose chest CT screening programs, the detection of ground-glass nodules (GGNs) is rapidly increasing. Early-stage lung adenocarcinomas (LUADs) often manifest as pure ground-glass nodules (GGNs) and part-solid nodules (PSNs), and the prognosis is significantly related to pathological subtypes of LUADs (3, 4). Sublobar resection (including wedge resection and segmentectomy) could be considered for some stage I non-small cell lung cancer (NSCLC) patients with pre-invasive adenocarcinoma (adenocarcinoma in situ, AIS), minimally invasive adenocarcinoma (MIA), or lepidic predominant adenocarcinoma, owing to its favorable prognosis (5). However, some subtypes (solid, micropapillary, and complex glandular) of LUADs often have a poor prognosis (6), indicating the necessity of lobectomy for these patients. Therefore, the accurate pre-judgment of pathological subtypes and gradings would benefit the selection of surgery type, prognosis, and personalized postoperative follow-up of stage I LUADs.

Currently, many radiomics models have been developed to classify main histologic subtypes of lung cancer, such as the differentiation of non-small cell lung cancer (NSCLC) and small cell lung cancers (SCLC) (7), the classification of lung adenocarcinomas (ADC) and squamous cell carcinomas (SCC) (8), the differentiation of ADC, SCC, and SCLC (9). Of note, studies on LUADs also focused on the histologic subtype classification, and most studies simplified the problem by dividing LUADs into a 2-category classification (IAC; non-IAC) according to their invasiveness (10). In addition to the invasiveness, subtypes indicative of poor prognoses, such as the invasive mucinous adenocarcinoma (IMA), are still rarely included in classification studies, especially for stage IA LUADs. Additionally, although some reports studied the identification of high-grade LUADs via radiomics, the systematic stratification of IAC grades according to the 2020 new grade system from the International Association for the Study of Lung Cancer (IASLC) Pathology Committee (6) was yet to be explored.

In this study, we focused on patients with stage IA LUADs and aimed to develop two consecutive radiomics models for their non-invasive histologic subtype classification and grade stratification. Of note, Deep learning (DL)-based pre-annotation strategy and expansion annotation strategies were utilized to study the influence of ROIs delineation on the performance of radiomics. In combination with multiple machine learning algorithms, stable radiomics models were selected based on their performance on internal, independent, and external testing sets and further underwent subgroup analysis, validating their potential in supporting the clinical decisions in the era of precise and personal medicine.

## Materials and methods

The retrospective study was approved by the Institutional Reviewing Board (IRB) of Beijing Haidian Hospital and Peking University People’s Hospital and the informed consent was waived by IRBs since patient information was anonymized to ensure privacy.

### Study population

Patients who underwent chest surgery and were diagnosed with stage IA LUAD were enrolled from two medical centers for radiomics model development and external validation according to the following including and exclusion criteria. Three cohorts were eventually included from two hospitals and constitute three datasets, including development set, independent test set, and external test set.

**Figure 1:**
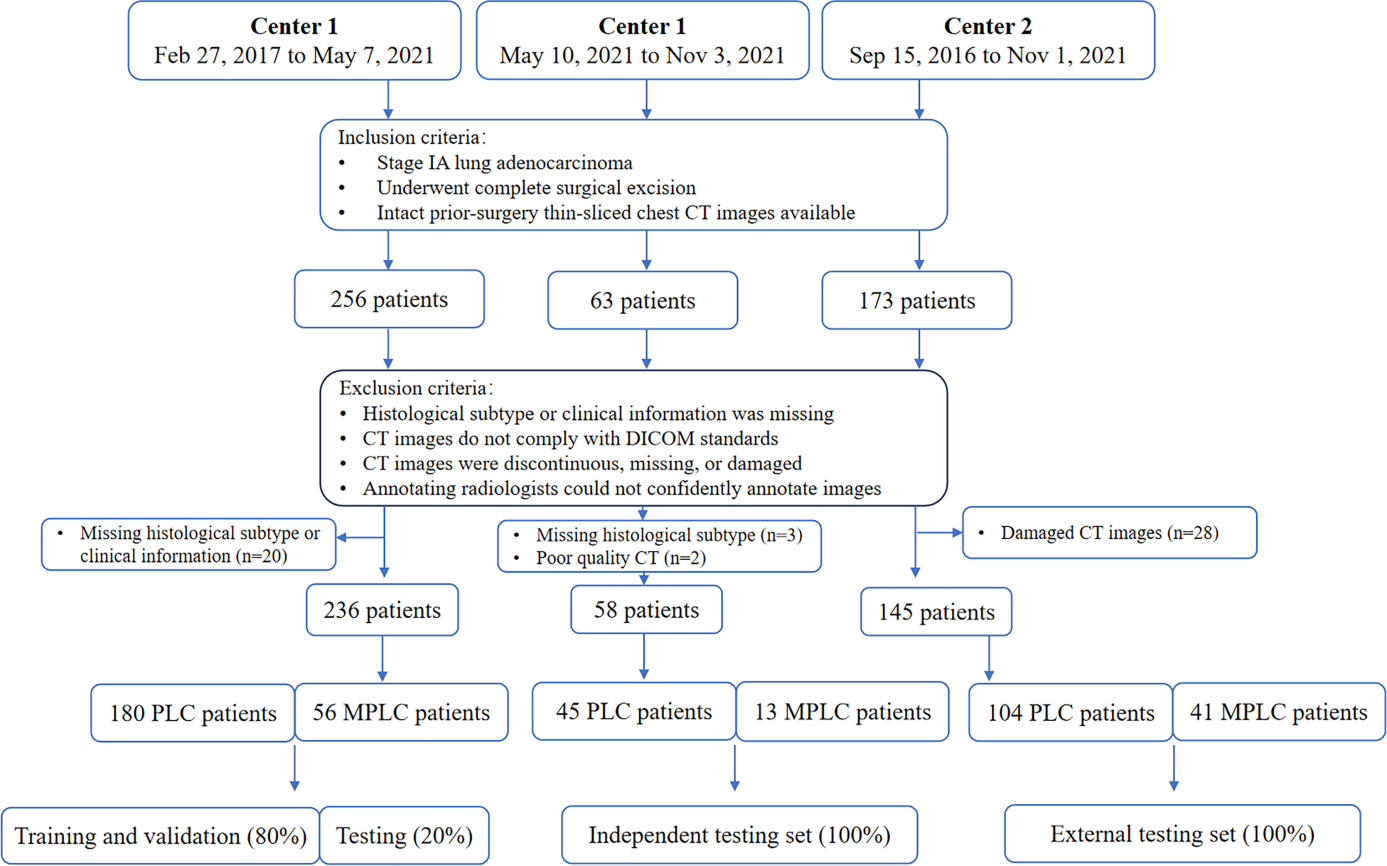
Diagram of patients enrollment and data partition. PLC = primary lung cancer, MPLC = multiple primary lung cancer, DICOM: Digital Imaging and Communications in Medicine.

The first cohort, comprising 236 patients treated at our institution between February 27, 2017, and May 7, 2021, included 180 primary lung cancer (PLC) patients with a single lesion and 56 multiple primary lung cancer (MPLC) patients. This dataset was used for radiomics development and was divided into training, validation, and internal testing subsets at a ratio of 16: 4: 5. The second cohort included 58 eligible patients treated between May 10, 2021, and Nov 3, 2021, and was used as an independent test set. Of note, to further evaluate the robustness and generalization of proposed radiomics models, 145 eligible patients who underwent treatment at the other hospital between Sep 15, 2016, and Nov 1, 2021, were enrolled in cohort 3 and served as the external test set. Diagrams of patient enrollment and data partition details can be found in **Figure 1**.

The inclusion criteria were as follows: *a*) patients with stage IA lung adenocarcinoma; *b*) those who underwent complete surgical excision; *c*) those with preoperative thin-sliced chest CT images. Patients were excluded if *a*) histological subtype or clinical information was missing; *b*) their CT images were not in compliance with the Digital Imaging and Communications in Medicine (DICOM) standards; *c*) CT images were discontinuous, missing, or damaged; *d*) annotating radiologists could not confidently annotate images.

### CT acquisition

All the enrolled patients underwent chest CT examinations before surgical excision. Particularly, multi-slice spiral CT low-dose scans were performed using instruments from GE Healthcare (Chicago, Ill, USA), Philips Healthcare (Amsterdam, Netherlands), and United Imaging (Shanghai, China). The key scanning parameters were as follows: tube voltage of 120KV; reconstruction slice thickness from 0.625 to 2mm. All CT scans were saved in the picture archiving and communication system.

### Deep learning segmentation algorithm-aided annotation of pulmonary nodules

Given that deep learning (DL)-based auxiliary diagnosis systems for pulmonary nodules have been well developed and launched in clinical settings (11, 12), a modified Faster R-CNN model trained on more than 11,000 chest CT scans to detect different types of pulmonary nodules was utilized to aid the annotation of targeted nodules (12). Briefly, the employed modified Faster R-CNN first detected the targeted nodules and a U-Net segmentation algorithm output the contour. Then, senior radiologists further corrected the delineation of interested pulmonary nodules and deleted untargeted nodule lesions. In such a way, consumption of the medical labor force was significantly reduced, and the annotation efficiency was greatly improved. The credibility of the DL-based segmentation algorithm in annotating pulmonary nodules was examined by comparing it with manual-corrected lesion contours.

### Expansion strategies for ROI annotation

Previous studies revealed that peritumoral information could improve the model performance on invasiveness prediction of ADC (13) and histological subtype stratification in patients with NSCLC (14). Another previous radiomics studies reported that bounding-box delineation of ROI could achieve equivalent performance to precisely annotated ones (15). Considering the potential advantages of peritumoral areas in histologic classification tasks, in addition to the DL-aided manual-correction annotation strategy, we further explored the pixel-expansion annotation strategy for radiomics modeling by expanding lesion contours based on manual corrected ones. Particularly, we performed 1-pixel, 3-pixel, 5-pixel, and bounding-box expansions after the manual correction was completed. The representation of annotated lesions was presented in the **Supplementary Figure 1**. Summarily, we selected different ROIs in this study, encompassing the precise lesion ROI, the expanded ROI, and the bounding-box ROI of the designated lesions. To ensure the accurate localization of the targeted lesion on CT images, a multidisciplinary team consisting of radiologists, thoracic surgeons, and pathologists collaborated in defining the targeted lesions. The impact of different annotation strategies on stage IA LUAD histologic subtype classification and invasive non-mucinous adenocarcinoma (IAC) grade stratification was analyzed in this study by comparing the performance of radiomics models.

### Feature extraction

The PyRadiomics package (version 2.2.0) was called using Python (version 3.8.1) when performing radiomics feature extraction. Summarily, a total of 1454 features were extracted from the annotated ROIs, which belonged to 7 classes, including first-order (FOS), shape, Gray Level Co-occurrence Matrix (GLCM), Gray Level Run Length Matrix (GLRLM), Gray Level Size Zone Matrix (GLSZM), Neighbouring Gray Tone Difference Matrix (NGTDM), and Gray Level Dependence Matrix (GLDM) features. Detailed information on extracted features was summarized in **supplementary table 1**.

**Table 1:** Clinical characteristics of enrolled patients.

Groups Information	Model development set			Independent test set			External test set			Test
	Overall	PLC	MPLC	Overall	PLC	MPLC	Overall	PLC	MPLC	p value
Patients*	236	180	56	58	46	12	145	104	41	
Age (y)	57.0 (29 ~ 83)	56.6 (29 ~ 83)	58.2 (39 ~ 79)	59.8 (34 ~ 84)	60.2 (39 ~ 84)	57.9 (34 ~ 76)	61 (54~66)	61 (55~66)	61 (52~66)	0.018
Sex										0.086
Female	158 (66.95%)	119 (66.11%)	39 (69.64%)	41 (70.69%)	32 (69.57%)	9 (75%)	83 (57.24%)	62 (59.62%)	21 (51.22%)	
Male	78 (33.05%)	61 (33.89%)	17 (30.36%)	17 (29.31%)	14 (30.43%)	3 (25%)	62 (42.76%)	42 (40.38%)	20 (48.78%)	
Smoking history										0.849
Current	33 (13.98%)	27 (15%)	6 (10.71%)	6 (10.34%)	3 (6.52%)	3 (25%)	17 (11.72%)	11 (10.58%)	6 (14.63%)	
Former	18 (7.63%)	13 (7.22%)	5 (8.93%)	4 (6.9%)	4 (8.7%)	0 (0%)	14 (9.66%)	10 (9.62%)	4 (9.76%)	
Never	185 (78.39%)	140 (77.78%)	45 (80.36%)	48 (82.76%)	39 (84.78%)	9 (75%)	114 (78.62%)	83 (79.81%)	31 (75.61%)	
Family history of cancer										<0.010
Yes	64 (27.12%)	46 (25.56%)	18 (32.14%)	18 (31.03%)	15 (32.61%)	3 (25%)	19 (13.1%)	13 (12.5%)	6 (14.63%)	
No	172 (72.88%)	134 (74.44%)	38 (67.86%)	40 (68.97%)	31 (67.39%)	9 (75%)	126 (86.9%)	91 (87.5%)	35 (85.37%)	
Alcohol intake history										0.743
Yes	33 (13.98%)	25 (13.89%)	8 (14.29%)	10 (17.24%)	8 (17.39%)	2 (16.67%)	19 (13.1%)	15 (14.42%)	4 (9.76%)	
No	203 (86.02%)	155 (86.11%)	48 (85.71%)	48 (82.76%)	38 (82.61%)	10 (83.33%)	126 (86.9%)	89 (85.58%)	37 (90.24%)	
Lesion†	308	180	128	76	46	30	221	104	117	
Nodule type by density										<0.010
GGN	94 (30.52%)	38 (21.11%)	56 (43.75%)	41 (53.95%)	21 (45.65%)	20 (66.67%)	50 (22.62%)	4 (3.85%)	46 (39.32%)	
mGGN	175 (56.82%)	113 (62.78%)	62 (48.44%)	27 (35.53%)	21 (45.65%)	6 (20%)	120 (54.3%)	65 (62.5%)	55 (47.01%)	
Solid	34 (11.04%)	25 (13.89%)	9 (7.03%)	8 (10.53%)	4 (8.7%)	4 (13.33%)	31 (14.03%)	21 (20.19%)	10 (8.55%)	
mass	5 (1.62%)	4 (2.22%)	1 (0.78%)	0 (0%)	0 (0%)	0 (0%)	20 (9.05%)	14 (13.46%)	6 (5.13%)	
Histologic subtypes										<0.010
AIS	17 (5.52%)	5 (2.78%)	12 (9.38%)	0 (0%)	0 (0%)	0 (0%)	11 (4.98%)	1 (0.96%)	10 (8.55%)	
AAH	8 (2.6%)	0 (0%)	8 (6.25%)	0 (0%)	0 (0%)	0 (0%)	9 (4.07%)	0 (0%)	9 (7.69%)	
MIA	100 (32.47%)	52 (28.89%)	48 (37.50%)	36 (47.37%)	18 (39.13%)	18 (60%)	51 (23.08%)	8 (7.69%)	43 (36.75%)	
IAC	169 (54.87%)	111 (61.67%)	58 (45.31%)	40 (52.63%)	28 (60.87%)	12 (40%)	141 (63.8%)	87 (83.65%)	54 (46.15%)	
IMA	14 (4.55%)	12 (6.67%)	2 (1.56%)	0 (0%)	0 (0%)	0 (0%)	9 (4.07%)	8 (7.69%)	1 (0.85%)	
IAC gradings										0.027
Grade 1	16 (9.47%)	11 (9.91%)	5 (8.62%)	9 (22.5%)	4 (14.29%)	5 (41.67%)	12 (8.51%)	11 (12.64%)	1 (1.85%)	
Grade 2	148 (87.57%)	95 (85.59%)	53 (91.38%)	30 (75%)	23 (82.14%)	7 (58.33%)	118 (83.69%)	69 (79.31%)	49 (90.74%)	
Grade 3	5 (2.96%)	5 (4.5%)	0 (0%)	1 (2.5%)	1 (3.57%)	0 (0%)	11 (7.8%)	7 (8.05%)	4 (7.41%)	

Unless otherwise indicated, data are numbers of patients. AIS, adenocarcinoma in situ; AAH, atypical adenomatous hyperplasia; MIA, minimally invasive adenocarcinoma; IAC, invasive non-mucinous adenocarcinoma; IMA, invasive mucinous adenocarcinoma; GGNs, ground-glass nodules; PLC, primary lung cancer; MPLC, multiple primary lung cancer.

^*^Data are the median, and data in parentheses are the interquartile range.

**^†^** Data are numbers of nodules.

### Dimension reduction of extracted radiomics features

Pearson correlation coefficient (PCC) was first calculated and used to reduce the redundancy of the primary feature set, followed by the principal component analysis (PCA) approach which converted potentially correlated features into principal components that are linearly uncorrelated via orthogonal transformation (16). Features with a PCC <0.8 were retained after the first-round examination of feature redundancy. Subsequently, uncorrelated principal feature components were further obtained via PCA and utilized to develop radiomics models for histologic subtype classification and IAC grade stratification. Feature selection was accomplished by calling the scikit-learn (version 0.20.2) package.

### Establishment of pathologic gold standard

Chest CT scans, pathological information, and clinical information was retrospectively collected from all included eligible patients and used to generate gold standard labels. Given the update of the WHO Classification of Tumors of the Lung in 2021 and IASLC grading system of IAC in 2020, histologic subtypes and IAC gradings of enrolled patients were all re-evaluated by an experienced pathologist before being utilized as the gold standard label in model development. In particular, histologic subtyping and grading were performed using the largest tumor sections in all cases, and the percentage of each histologic component was recorded in 5% increments according to the proposed IASLC grading system as follows: Grade 1, lepidic predominant tumors with no or less than 20% high-grade patterns (solid, micropapillary, and/or complex glandular patterns); Grade 2, acinar or papillary predominant tumors with no or less than 20% high-grade patterns; and Grade 3, any tumor with 20% or more of high-grade patterns.

### Development and evaluation of radiomics models

Based on the five ROI annotation strategies mentioned above, four classic machine learning (ML) algorithms were utilized to develop radiomics models, including support vector machine (SVM), logistic regression (LR), and multi-layer perceptron (MLP), and eXtreme Gradient Boosting (XGBoost). The optimal hyper-parameters of ML algorithms were determined by the model performance on the validation set. The stable ML algorithm and potential practical annotation strategy were explored according to the model performance on the test datasets.

**Figure 2:**
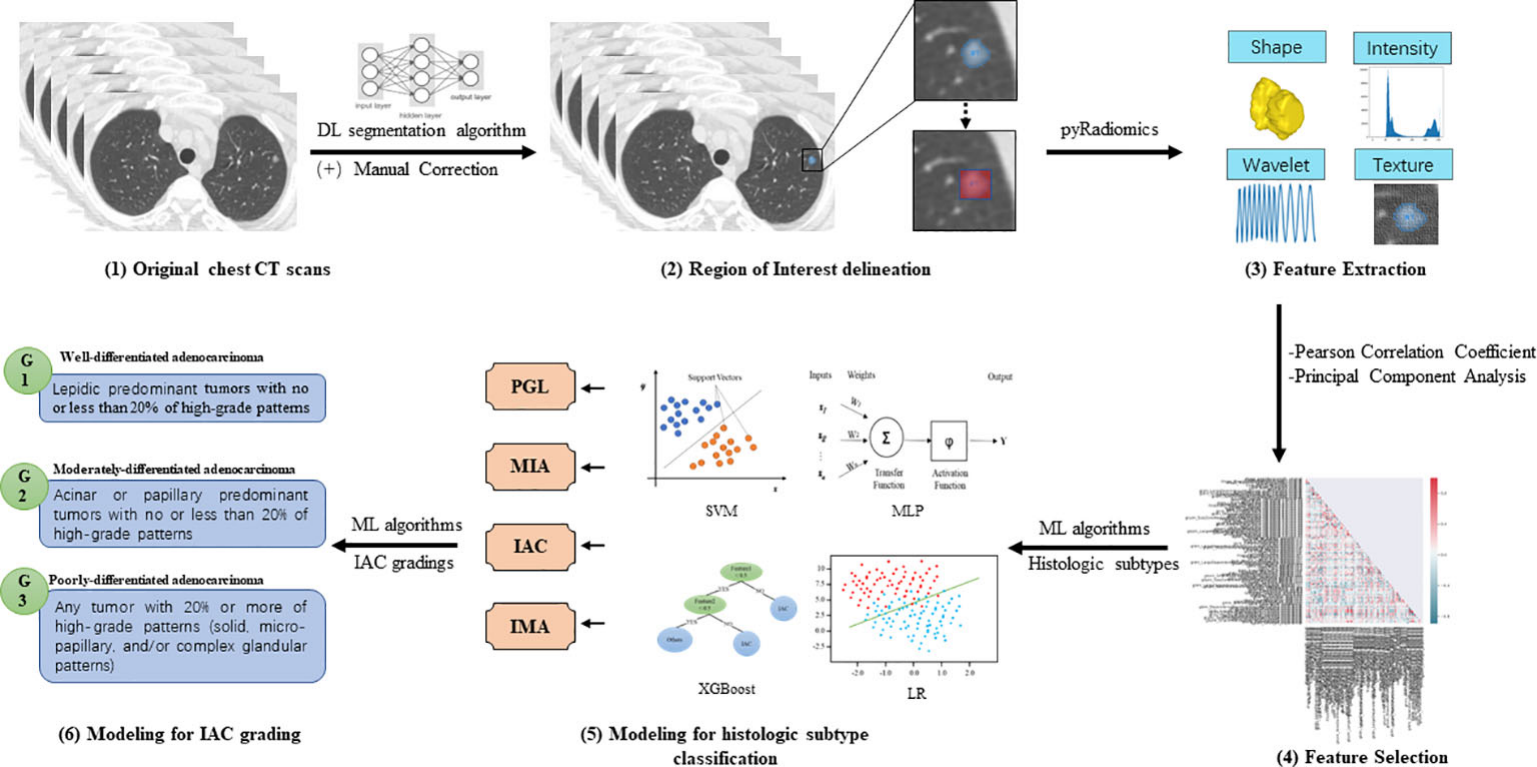
Illustration of the radiomics models for histologic subtype classification and IAC grading (1). Pre-operative chest CT scans were collected from enrolled patients for model development (2). Deep learning (DL)-based pulmonary nodule segmentation algorithm was utilized to pre-segment the target nodular lesions, followed by manual correction. Based on the manually edited region of interest (ROI), expansion strategies were applied to generate 1-pixel, 3-pixel, 5-pixel, and bounding-box masks of targeted lesions (3). PyRadiomics was utilized to extract radiomics features of different categories, including shape, intensity, wavelet, and texture features (4). Pearson correlation coefficient (PCC) and principal component analysis (PCA) were employed to reduce the dimensionality of extracted features (5). Classic machine learning (ML) algorithms were then used to develop radiomics models for classifying histologic subtypes of stage IA LUADs (6). Furthermore, ML algorithms were used to develop radiomics models for stratifying grades of invasive non-mucinous adenocarcinoma (IAC).

Radiomics models’ performance was evaluated by classification sensitivity, specificity, precision, accuracy, F1 score, G-Mean, and area under the ROC curve (AUC). According to the study design, the first batch radiomics models focused on the classification of stage IA LUAD histological subtype classification, including precursor glandular lesions (PGL), MIA, IAC, and IMA. The second batch radiomics models were responsible for the stratification of IAC grade (6), which ranged from grade 1 to grade 3 (**Figure 2**).

### Statistical analysis

Continuous variables were represented by the means ± SD while the categorical variables were expressed in terms of frequency and statistically analyzed by the Chi-square test. P <0.05 was considered statistically significant. A two-sided 95% confidence interval for AUC was constructed following the approach of Hanley and McNeil (1982) (17). Cohen’s Kappa coefficient was calculated in a confusion matrix to measure the agreement between pathological gold-standard and model predictions. All statistical analyses were performed with the R statistical package (The R Foundation for Statistical Computing, Vienna, Austria).

## Results

### Patient characteristics

From the two institutions, 256, 63, and 173 patients were initially eligible for the development set, independent test set, and external test set, respectively. However, due to missing histological subtype or clinical information, 20 (7.8%) and 3 (4.7%) patients were excluded. Additionally, 2 (3.2%) patients with motion-artifact induced poor quality CT scans and 28 (16.2%) patients with damaged CT scans were omitted. Thus, the final sample comprised 236, 58, and 145 patients in the development set, independent test set, and external test set (**Figure 1**).

In general, most of the included patients (79.04%, n=347) were non-smokers. Current (12.76%, n=56) and former smokers (8.20%, n=36) just count for a small portion of the studied population. Of note, 23.01% (n=101) of the population had a family history of cancer while 14.12% (n=62) of them had an alcohol intake history. The most frequent surgical procedure was lobectomy (38.95%, n=171), followed by segmentectomy (26.65%, n=117) and wedge resection (21.41%, n=94); the rest of included patients (12.98%, n=57) received hybrid surgical procedures due to the presence of multiple primary lung cancer lesions. At adenocarcinoma lesion level, most of them presented as PSNs (53.22%, n=322), followed by GGN (30.58%, n=185), solid nodule (12.07%, n=73), and mass (4.13%, n=25). With respect to histologic subtypes, IAC (57.85%, n=350), MIA (30.91%, n=187), PGL (7.44%, n=45), and IMA (3.80%, n=23) were included. Additionally, most IAC lesions (84.57%, n=296) were categorized as Grade 2 according to the latest released grading system by the IASLC Pathology Committee.

Detailed characteristics of the included population in different datasets was summarized in **Table 1**. Notably, patients in the external test set were significantly older than those in the development set. Furthermore, family history of cancer was significantly less common among patients in external test set. It is also worth noting that the distribution of nodule types by density, histologic subtypes, and IAC gradings significantly varied across datasets due to different data collection timeframes. Notably, the independent test set lacked PGL and IMA lesions.

### Analysis of radiomics features

A total of 1454 features were extracted from the annotated ROIs. A sum of 303 features with a Pearson correlation coefficient <0.8 was obtained after the first-round reduction of feature dimensionality. The correlation heatmap of selected features was presented in **Supplementary Figure 2A**. Subsequently, 40 principal feature components were preserved via PCA for the development of radiomics models. Principal component contribution rate was displayed in **Supplementary Figure 2B**. Details information about the extracted and selected features can be found in **Supplementary Table 1**.

**Figure 3:**
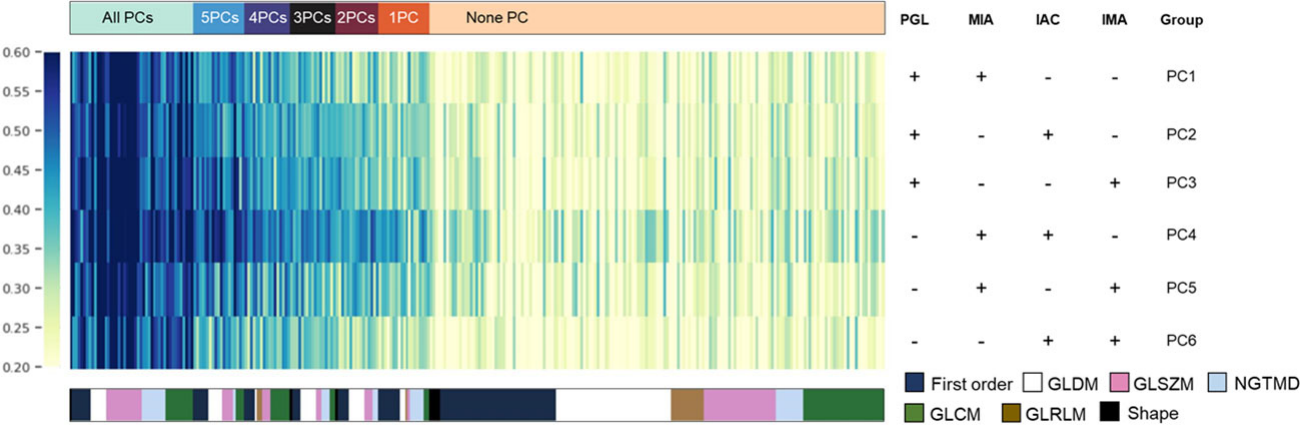
The most discriminative features for each histologic subtype. Based on PCC dimensionality reduction, distinguished features in a pair-wise comparison were analyzed to explain the potential key factors that distinguish them from each other. The detailed composition of each pair-wise comparison in each row is indicated in the right panel. Features were color-coded according to their category and listed from left to right based on their frequencies in pair-wise comparisons.

Since PCA analysis selected feature components rather than certain features, we analyzed the significantly distinguished features (SDF) between each subtype based on PCC selected features in advance before developing the four-class histologic subtypes classification model and obtained 6 pairwise comparisons (PCs). Of the first-round selected 303 features, SDFs between each subtype were identified and grouped according to their identifying frequencies. Features were eventually divided into 7 groups, including SDFs in all PCs (n=46), 5PCs (n=19), 4PCs (n=17), 3PCs (n=17), 2PCs (n=16), 1PC (n=19), and none of the 6 PCs (n=169). These divided feature groups and their corresponding categories were displayed in the feature heatmap (**Figure 3**), and the details of features in each group were listed in **Supplementary Table 2**.

### Selection of the optimal radiomics models for histologic subtypes classification and IAC grade stratification

**Figure 4:**
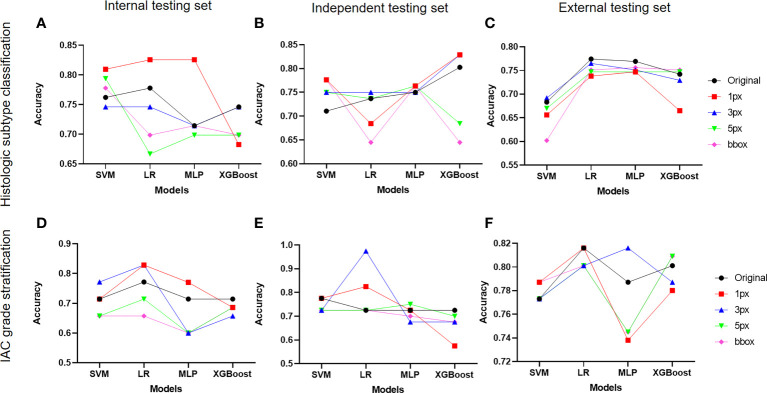
Impact of different annotation strategies on radiomics model performance. The performance of radiomics models developed on features from different annotation strategies were evaluated and compared in terms of accuracy. **(A-C)** displayed the accuracy of radiomics models for histologic subtype classification on the internal, independent, and external testing sets, respectively. **(D–F)** demonstrated the accuracy of radiomics models for IAC grade stratification on the internal, independent, and external testing sets.

DL-based nodule segmentation algorithms have enhanced the practicality of radiomics models. In the current study, we further employed five annotation strategies and four ML algorithms to develop two batches of models for LUAD diagnosis, including histologic subtype classification and IAC grade stratification. We first selected the optimal ML algorithms for both tasks by comparing the models’ performance under different annotation strategies on three test sets. As depicted in **Figures 4A–C**, MLP with 1-pixel annotation exhibited optimal performance on histologic subtype classification on the internal test set, and maintained consistent and excellent performance on independent and external test sets, regardless of annotation strategies. Notably, the bounding-box annotation strategy yielded comparable results for histologic subtype classification on the independent and external sets. Concurrently, LR displayed an overall superior performance on IAC grade stratification in terms of accuracy (**Figures 4D–F**). However, the performance of LR varied with different annotation strategies for IAC grade stratification.

Subsequently, impacts of annotations on selected ML algorithms were further evaluated on three test sets in terms of AUC, sensitivity, specificity, precision, F1-score, and G-Mean (**Supplementary Figure 3**). It was observed that MLP for histologic subtype classification had no preference for a specific annotation strategy, while LR for IAC grade stratification showed a preference for certain data labeling strategies. Regarding the performance of the radiomics models on each class, we noted inferior results for those classes with insufficient sample sizes.

### Performance evaluation of selected radiomics model for histologic subtypes classification

**Figure 5:**
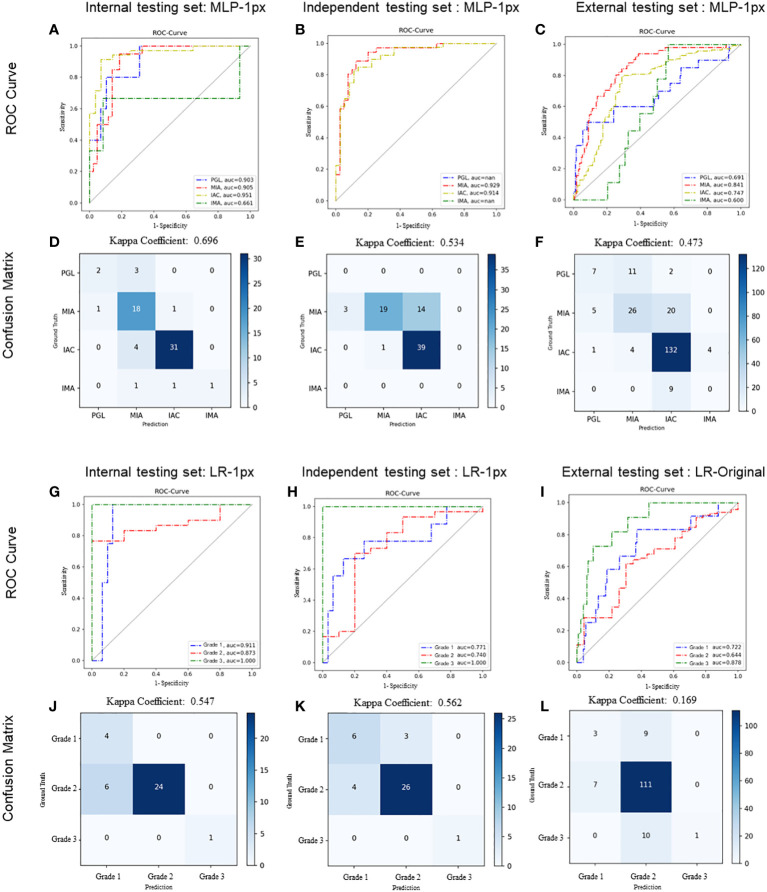
Performance of radiomics models on histologic subtype classification and IAC grading stratification. For histologic subtype classification, ROC curves were plotted to evaluate the performance of the Radiomic model in discriminating PGL, MIA, IAC, and IMA from the other three categories on internal **(A)**, independent **(B)**, and external **(C)** testing sets, respectively. Confusion matrices for four-category classification of PGL, MIA, IAC, and IM on internal **(D)**, independent **(E)**, and external **(F)** testing sets, respectively. For IAC grading stratification, ROC curves were plotted to evaluate the performance of the Radiomic model on internal **(G)**, independent **(H)**, and external **(I)** testing sets, respectively. Confusion matrices for the stratification of IAC grades (grade 1 to 3) on internal **(J)**, independent **(K)**, and external **(L)** testing sets, respectively. The exact number of true positives, false positives, true negatives and false negatives were listed. Kappa coefficients were calculated.

We first evaluated the performance of the radiomics model on histologic subtype classification. The MLP with 1-pixel expansion was selected as the representative model. This model achieved an AUC of 0.903, 0.905, 0.951, and 0.661 for PGL, MIA, IAC, and IMA lesions, respectively, on the internal test set. On the external test set, it achieved an AUC of 0.929 and 0.914 for MIA and IAC lesions. On the external test set, it achieved an AUC of 0.691, 0.841,0.747, and 0.600 for PGL, MIA, IAC, and IMA lesions, respectively (**Figures 5A–C**). Notably, the performance of MLP was compromised on the external test set. Meanwhile, the kappa coefficient of MLP reached 0.696, 0.534, and 0.473, which presented a substantial and moderate agreement between model-predicted histologic subtypes and ground truth (**Figures 5D–F**). A decrease in the accuracy of MLP was also observed among the internal, independent, and external test sets (**Table 2**). This discrepancy could potentially be attributed to the prevalence of challenging GGN lesions in the independent set and MPLC lesions in the external set. Of note, the accuracy of MLP remained stable on the external test sets (0.714 vs. 0.763 vs. 0.756) when the bounding-box annotation strategy was applied. The detailed performance metrics were summarized in **Table 2**.

**Table 2:** Detailed diagnostic metrics of radiomics models on internal, independent, and external test datasets.

Task	Test Sets	Subtypes/Grades	Accuracy (95%CI)	AUC (95%CI)	Sensitivity	Precision	Specificity	F1-Score	G-Mean
Histological subtypes	Internal	PGL	0.825 (0.730-0.921)	0.903 (0.712-1.000)	0.400	0.667	0.983	0.500	0.516
		MIA		0.905 (0.824-0.970)	0.900	0.692	0.814	0.783	0.789
		IAC		0.951 (0.890-0.994)	0.886	0.939	0.929	0.912	0.912
		IMA		0.661 (0.049-1.000)	0.333	1.000	1.000	0.500	0.577
		Average		0.855	0.630	0.825	0.932	0.674	0.699
	Independent	PGL	0.763 (0.659-0.855)	-	-	-	0.961	-	-
		MIA		0.929 (0.863-0.978)	0.528	0.950	0.975	0.679	0.708
		IAC		0.914 (0.846-0.971)	0.975	0.736	0.611	0.839	0.847
		IMA		-	-	-	0.987	-	-
		Average		0.922	0.752	0.843	0.884	0.759	0.778
	External	PGL	0.747 (0.688-0.805)	0.691 (0.534-0.826)	0.350	0.538	0.970	0.424	0.434
		MIA		0.841 (0.779-0.894)	0.510	0.634	0.912	0.565	0.569
		IAC		0.747 (0.676-0.814)	0.936	0.810	0.612	0.868	0.870
		IMA		0.600 (0.494-0.712)	0.000	0.000	0.981	0.000	0.000
		Average		0.720	0.449	0.496	0.869	0.464	0.468
IAC grading	Internal	Grade 1	0.829 (0.714-0.943)	0.911 (0.750-1.000)	1.000	0.400	0.807	0.571	0.633
		Grade 2		0.873 (0.735-0.983)	0.800	1.000	1.000	0.889	0.894
		Grade 3		1.000 (0.500-1.000)	1.000	1.000	1.000	1.000	1.000
		Average		0.928	0.933	0.800	0.936	0.820	0.842
	Independent	Grade 1	0.825 (0.700-0.925)	0.771(0.553-0.943)	0.667	0.600	0.871	0.632	0.633
		Grade 2		0.740 (0.516-0.935)	0.867	0.897	0.700	0.881	0.882
		Grade 3		1.000 (0.500-1.000)	1.000	1.000	1.000	1.000	1.000
		Average		0.837	0.845	0.832	0.857	0.838	0.838
	External	Grade 1	0.816 (0.752-0.879)	0.722 (0.566-0.860)	0.250	0.300	0.946	0.273	0.274
		Grade 2		0.644 (0.512-0.756)	0.941	0.854	0.174	0.895	0.896
		Grade 3		0.878 (0.764-0.953)	0.090	1.000	1.000	0.167	0.302
		Average		0.748	0.427	0.718	0.707	0.445	0.491

Data in parentheses are 95% CIs. AUC, area under the receiver operating characteristic curve; PGL, precursor glandular lesions; MIA, minimally invasive adenocarcinoma; IAC, invasive non-mucinous adenocarcinoma; IMA, invasive mucinous adenocarcinoma.

“-” means not applicable (N/A).

### Performance evaluation of optimal radiomics model for IAC grade stratification

We next evaluated the performance of the selected LR with optimal annotation strategies for IAC grade stratification. The LR model achieved an AUC of 0.911, 0.873, and 1.000 for grade 1, grade 2, and grade 3, respectively, on the internal testing set (**Figure 5G**), with a corresponding kappa coefficient of 0.547 (**Figure 5J**). However, on the independent test set, the LR model yielded a lower AUC of 0.771, 0.740, and 1.000 for grade 1, grade 2, and grade 3 respectively, and on the external test set, an AUC of 0.772, 0.644, and 0.878 for grade 1, grade 2, and grade 3, respectively. This suboptimal performance could be attributed to the imbalanced in sample size across the different grades (**Figures 5H, I**). The kappa coefficients of the LR model on the independent and external sets were 0.562 and 0.169, respectively (**Figures 5K, L**). Detailed performance metrics were summarized in **Table 2**.

### Subgroup analysis of selected representative ML model performance on test sets

**Figure 6:**
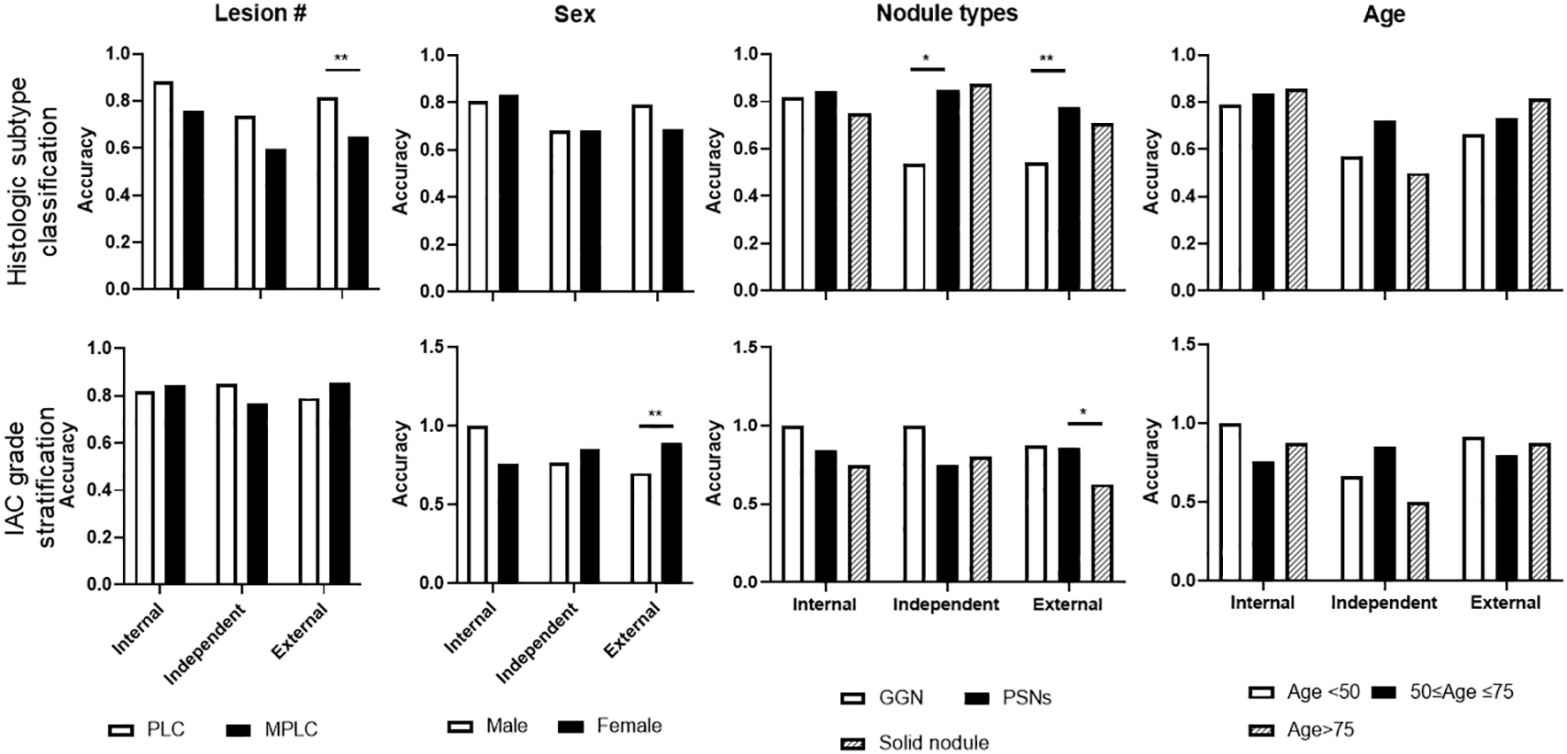
Subgroup analysis of selected representative ML model performance on test sets. Subgroup analyses were performed on histologic subtype classification and IAC grade stratification on internal, independent, and external testing sets, including target lesion numbers per patient, sex, nodule types, and age periods.

Notably, subgroup analyses of lesion numbers (PLC Vs MPLC), sex, nodule types by density (GGN vs PSNs vs solid), and age range were further performed (**Figure 6**). For histologic subtype classification, lower accuracy of MLP were observed on MPLC patients, significantly lower level was found on external test sets. Besides, significantly lower accuracy of MLP was also seen in GGN lesions on independent and external test sets. For IAC grade stratification, LR displayed significantly lower accuracy on male patients and solid nodules on the external test set. No significant difference of both two models was observed among other subgroups.

## Discussion

Non-invasive preoperative prediction of pathological subtype and grade would greatly benefit the patients with stage IA LUADs in terms of the selection of surgery type, prognosis, and personalized postoperative follow-up. In this current study, we proposed two consecutive radiomics models for the diagnosis of patients with LUADs, including histologic subtype classification (PGL, MIA, IAC, and IMA) and IAC grade stratification (grade 1-3). Five annotation strategies and four ML algorithms were utilized for modeling. MLP and LR were selected as the optimal algorithms for histologic subtype classification and IAC grading stratification tasks, respectively, as supported by the overall better performance on different annotations on internal, independent, and external test sets. For histologic subtype classification, bounding-box annotation enabled an equivalent performance of MLP. Besides, distinguishing features between each pairwise comparison were revealed. Additionally, subgroup analyses validated the applicability of the radiomics models across cohorts with different sex, ages, and number of lesions.

Radiomics has been used since 2014 to solve clinical problems (18), and as its applications expand, efforts to streamline the process for clinical implementation are ongoing. Lesion annotation is often time-consuming and labor-intensive, limiting the clinical deployment of radiomics tools. Previous studies (19, 20) reported that semiautomatic lesion segmentation exhibited high agreement with manual delineations and could provide a significant reduction in interobserver variability. Some other studies utilized certain whole CT images (21), certain annotated slides (22), or bounding-box annotation (15) to develop models which could also avoid heavy annotation workload but might result in insufficient features. Given that DL segmentation algorithms for pulmonary nodules were well trained (11, 12), we then employed one to pre-segment the targeted lesions followed by a manual edition. The employed DL algorithm achieved an averaged Dice index of 0.94 (compared with manually edited contours), indicating the potential of the end-to-end or enhanced radiomics models by integrating DL segmentation algorithms into the classic radiomics modeling pathway. However, unlike the DL-enable end-to-end radiomics model in differentiating COVID-19 (22), we enrolled MPLC patients with other untargeted nodules that needed to be manually excluded before developing radiomics models. After all, as previously reported (23, 24), our hybrid approach avoided intensive labor force for lesion annotation.

Since the easy-to-use bounding box annotation strategy was proved to be efficient in developing radiomics models for the diagnoses of gastric cancer and breast lesions (15, 25), we also examined the efficiency of an expansion strategy for the LUADs related tasks in our study by generating 1, 3, 5-pixel expanded and bounding-box (based on 5-pixel expansion) annotations. Notably, the 1-pixel expansion strategy, to some extent, enabled an overall stable performance of selected ML algorithms. An expansion strategy on cancerous lesions seemed to be a good option to enhance the model performance possibly by including more peritumoral features. Of course, the degree of expansion will need to be determined according to the situation. For histologic subtype classification, although the 1-pixel expansion strategy enabled an overall better performance, we also noticed the accuracy decline of MLP algorithm from internal to external test sets. Of note, accuracy of MLP remained acceptable and stable among test sets when applying the bounding-box strategy, indicating the practicality of the bounding-box strategy in this histologic subtype classification task. In contrast, the bounding-box strategy didn’t perform well on the three-grade classification tasks in this study, indicating its applicability is algorithm- and context-dependent.

Another essential procedure for radiomics is dimensionality reduction which plays a key role in alleviating ML artifacts in the scenario of unbalanced datasets with small sample sizes (26). We utilized two classic approaches, PCC and PCA, to perform the dimensionality reduction in this study (27, 28). As an unsupervised method, PCA projected features into a dimensionally reduced set of uncorrelated variables called principal components via the linear orthogonal transformation, and outperformed the supervised technique in terms of generalizability capability (26). However, to solve the main problem of the variable’s interpretation loss, we analyzed the distinguished features in a pair-wise comparison after PCC-based dimensionality reduction. The significant discriminating features between pair-wise comparisons may explain, to some extent, the key factors that distinguish them from each other.

Most previous related radiomics studies focused on binary classification in distinguishing NSCLC from SCLC, ADC from SCC, and IAC from other less invasive LUADs (7, 8). Given the update of the WHO Classification of Tumors of the Lung in 2021 and IASLC grading system of IAC in 2020 and the unique manifestations of IMA, we developed the first radiomics models for identifying four-category subtypes (PGL, IMA, IAC, and IMA) and three-category grades (grade1 to 3). We employed 4 classic ML algorithms and found that MLP and LR displayed an overall stable performance for four-category subtypes and three-category grades tasks, respectively. With the respect to identifying multi-class histologic subtypes, the selected representative MLP model in the current study achieved an average AUC of 0.855 and 0.922 on internal and independent testing sets, outperforming other models with an average AUC of 0.747 (4-category of NSCLC) (29), 0.833 (3-category subtypes of central lung cancer) (9), and 0.896 (4-category subtype of AAH, AIS, MIA, and IA) (30) in previous studies. Notably, the multiclass histological subtype classification model was not externally tested in previous studies, whereas the MLP achieved a mean AUC of 0.720 on external test set in this study. Meanwhile, few studies have reported the radiomics approach to stratify IAC grades according to the newly updated grading system. Instead, the radiomics approach was used to predict the micropapillary pattern that was reported to have a poor prognosis in a previous study (31). In comparison to multiparametric MRI-based radiomics approach for NSCLC grading (AUC 0.767) and contrast-enhanced CT-based radiomics signature for prediction of tumor differentiation degree (low and high degree, AUC 0.782) (32, 33), the selected representative LR algorithm for IAC grade stratification in this study achieved better performance on both internal and independent testing sets (averaged AUC 0.928 and 0.837) and equivalent performance on external test set (averaged AUC 0.748), indicating the potential of CT-based radiomics approach in predicting histologic grades of IAC. Meanwhile, we noticed a dramatically decreased Kappa coefficient of LR algorithm on external test set, which caused by the miss classifications of grade1 and 3 into grade 2, suggesting the need of further improvement for IAC grading stratification algorithms by including more balanced data.

Of note, a previous study performed radiogenomic analyses of patients with stage I LUAD by an unsupervised consensus clustering approach to better classify patients with different prognoses, complementing the TNM system (34). In consistent, we developed supervised radiomic models on the patients with stage IA LUAD (not including IB) to enable the accurate differentiation of patients with poor prognosis at early stages according to histologic subtypes. To address the heterogeneity of LUAD, we further included the histologic type of IMA in the proposed model. IMA has different characteristics than non-mucinous adenocarcinoma in terms of histology, radiological and clinical features. Although IMA can show a lepidic growth pattern, invasive patterns are always present. Several studies have shown that IMA has a poor prognosis than non-mucinous adenocarcinoma (35–37). Additionally, IMA is commonly detected in the advanced stage and cannot be surgically treated. Therefore, our proposed radiomics models, to some extent, aided the accurate pre-judgment of patients’ prognoses. Furthermore, they validated the revealed associations between CT-based radiomic features and known prognostic histologic factors, genomic drivers, and patient outcomes in the solid-type subgroup. In our subgroup analysis, the accuracy for differentiating histologic subtypes between GGN and PSNs lesions on both independent and external test sets were found to be significantly different.

There are some limitations to our study. The imbalance in histologic subtypes in the dataset compromised the performance of our proposed classification models, especially for PGL and IMA subtypes, and grade 3 lesions, which were less common in patients with operable clinical stage IA lung adenocarcinoma in clinical practice. The short follow-up of enrolled patients limited our ability to investigate the associations between radiomics and clinical features and the prognosis of patients with clinical stage IA LUAD. Although it is difficult for doctors to precisely classify those subtypes and grades, future work is also necessary to reveal the auxiliary effect of both models in promoting the diagnostic capabilities of these histologic subtypes, especially the identification of IMA, and IAC grades.

Despite these limitations, our results suggest that radiomics model, represented by MLP and LR, have great potential to predict the fine histological subtypes and grades of early-stage LUADs based on CT images, potentially providing a promising noninvasive approach for the diagnosis and management of early-stage LUADs.

## Data availability statement

The raw data supporting the conclusions of this article will be made available by the authors, without undue reservation.

## Ethics statement

The studies involving human participants were reviewed and approved by The Institutional Reviewing Board of Beijing Haidian Hospital and Peking University People’s Hospital. Written informed consent for participation was not required for this study in accordance with the national legislation and the institutional requirements.

## Author contributions

YH designed the study and controlled the data used in this study. GP, KS, YY, QL, and SW participated in the collection of patients’ data and manual correction of ROIs and provided clinical expertise. DW, WT, YS, and SY were responsible for modeling and testing. KS was responsible for quality control of the pathological samples. GP and DW prepared the main manuscript text. YH further polished the manuscript. All authors reviewed the manuscript. All authors contributed to the article and approved the submitted version.
