# Supplementary material for: Deep learning-enhanced radiomics for histologic classification and grade stratification of stage IA lung adenocarcinoma: a multicenter study
Source: Front Oncol. 2023 Jul 20;13:1224455. doi: 10.3389/fonc.2023.1224455 (PMC10400286; doi:10.3389/fonc.2023.1224455)
Supplement: Supplementary file 1 [file Table_1.docx]

Supplementary Material

Deep Learning-Enhanced Radiomics for Histologic Classification and Grade Stratification of Stage IA Lung Adenocarcinoma: a multicenter study

**Guotian Pei^1^**^†^**, Dawei Wang^2^**^†^**, Kunkun Sun^3^, Yingshun Yang^1^, Wen Tang^2^, Yanfeng Sun^2^, Siyuan Yin^2^, Qiang Liu^1^, Shuai Wang^1^, Yuqing Huang^1^**^*^

*** Correspondence:**

*****Yuqing Huang，Email: [huangyuqing555@gmail.com](mailto:huangyuqing555@gmail.com)

## Supplementary Figures


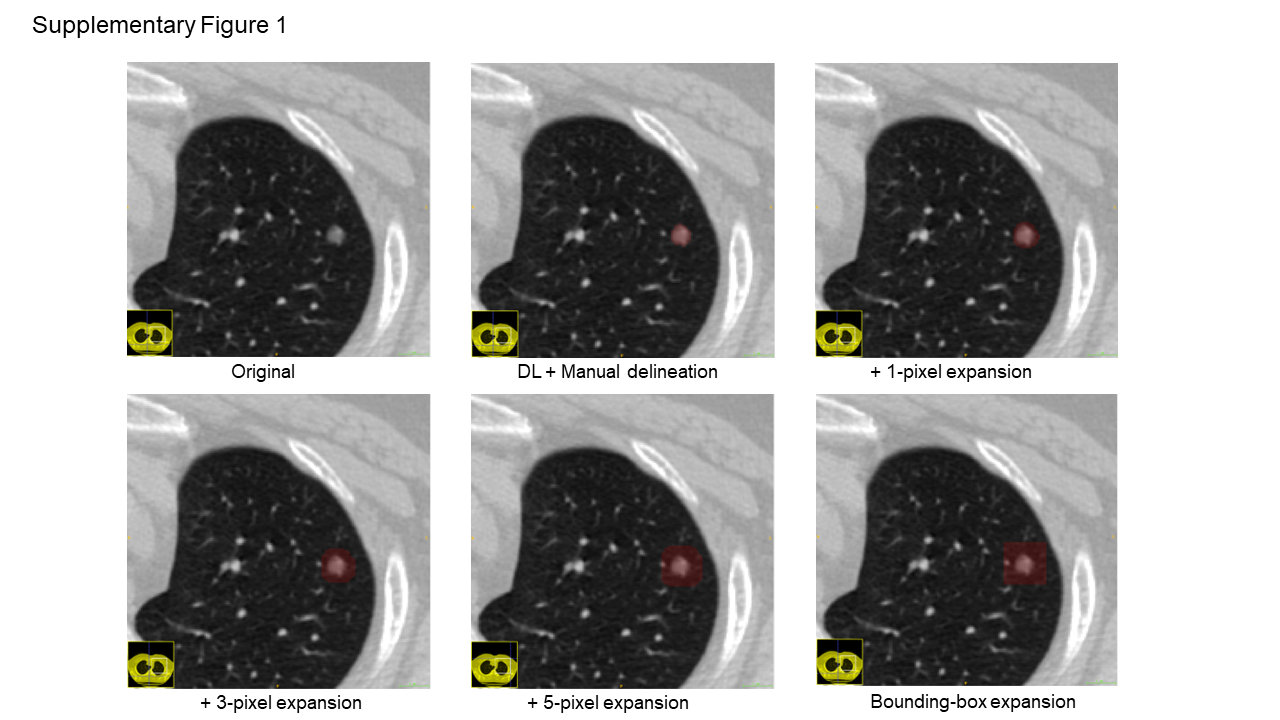


**Supplementary Figure 1. Representative of the delineation and expansion of ROIs.** Representatives of different annotation strategies were shown, including original chest CT image, DL-based manually edited delineation of ROI, 1-pixel expanded delineation, 3-pixel expanded delineation, 5-pixel expanded delineation, and bounding-box expanded delineation.


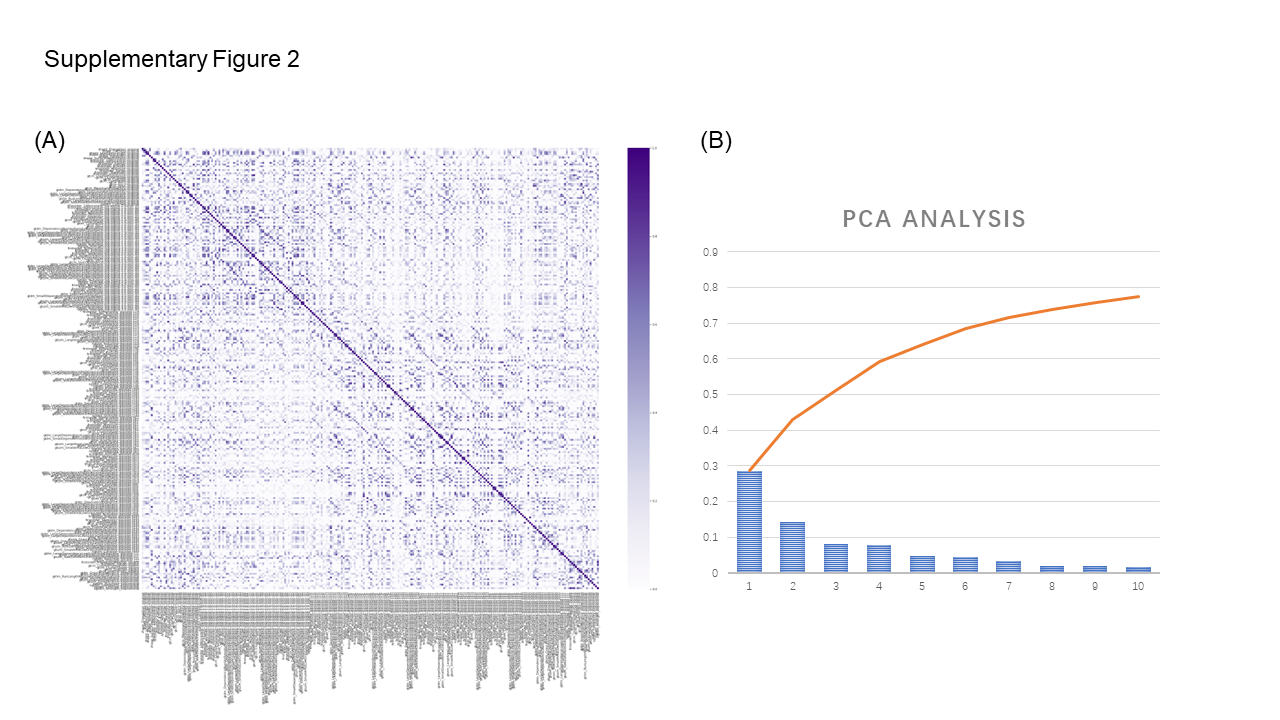


**Supplementary Figure 2. Screening the optimal feature selection method for coarse and fine models.**  Features with a Pearson correlation coefficient <0.8 was obtained after the first-round reduction of feature dimensionality. The correlation heatmap of selected features was presented in panel A. In the subsequent PCA analysis, the principal component contribution rate was displayed in panel B.


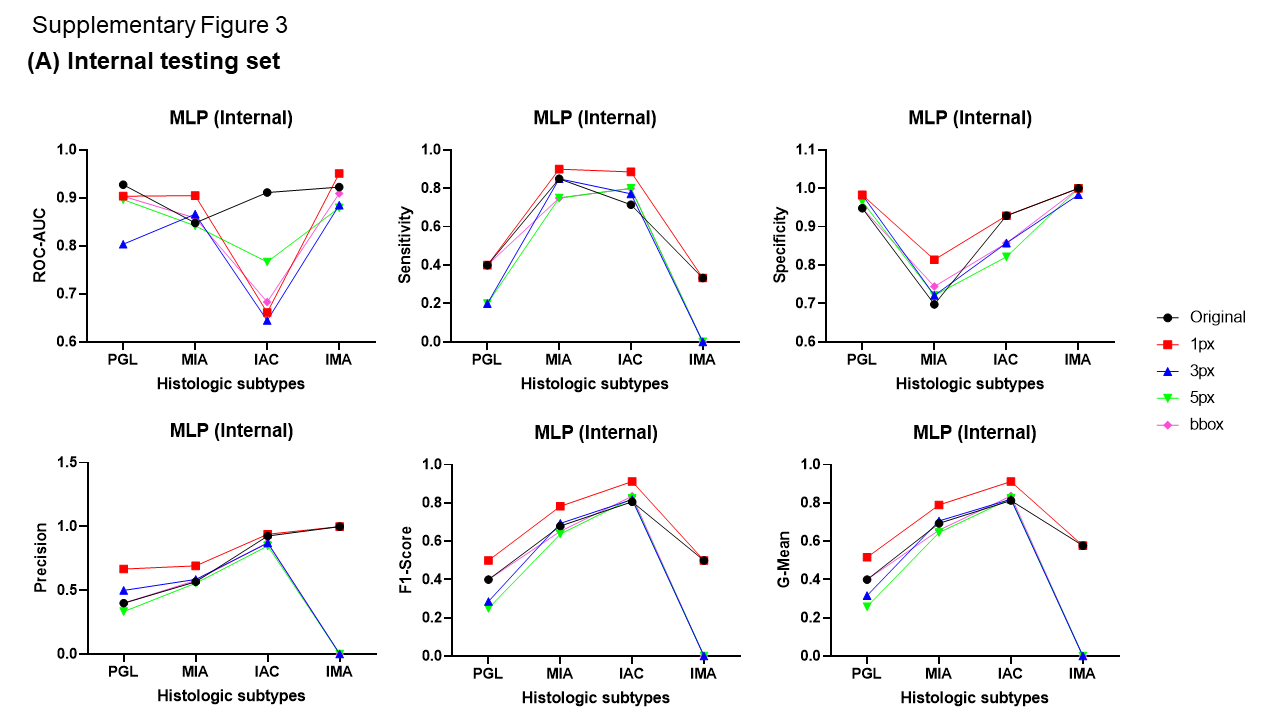


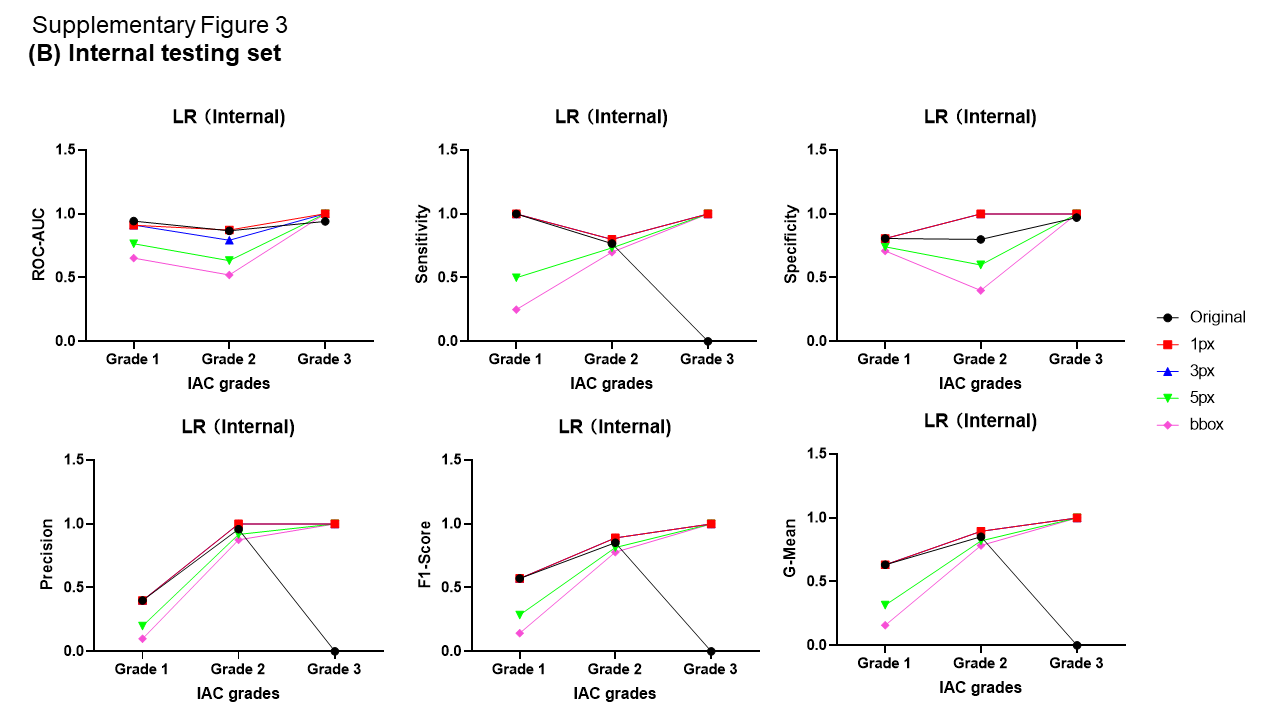


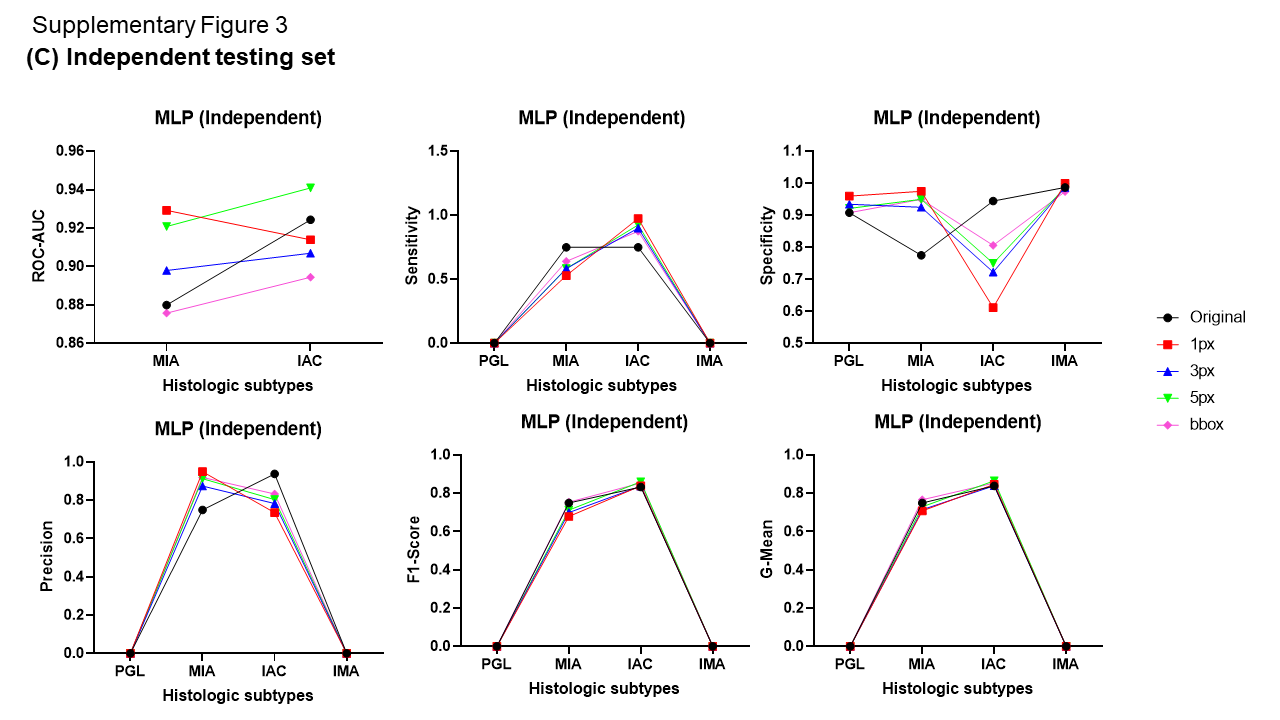


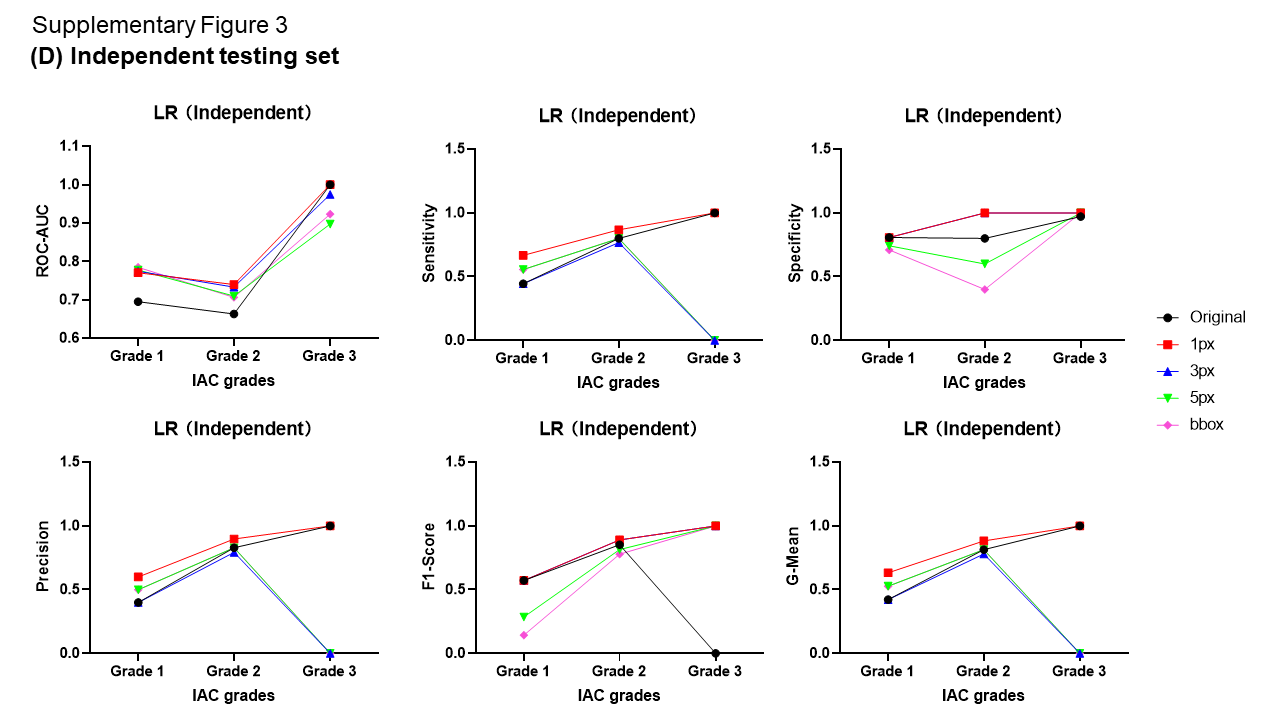


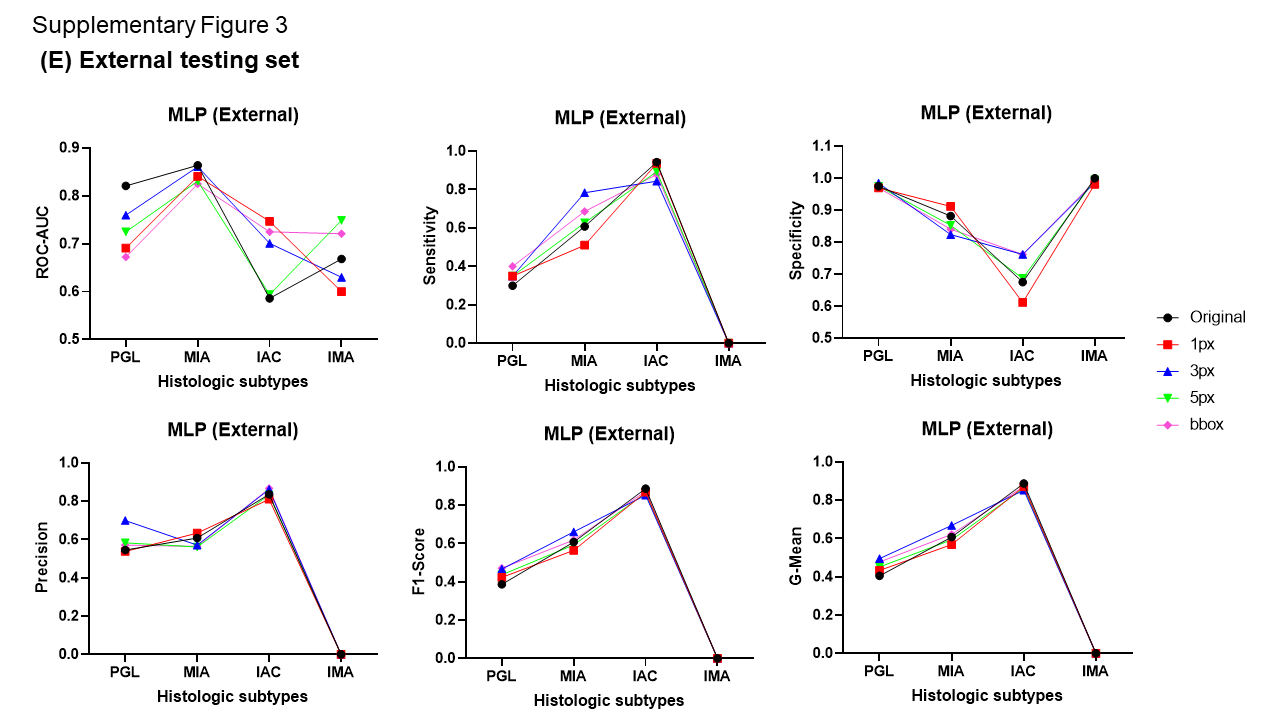


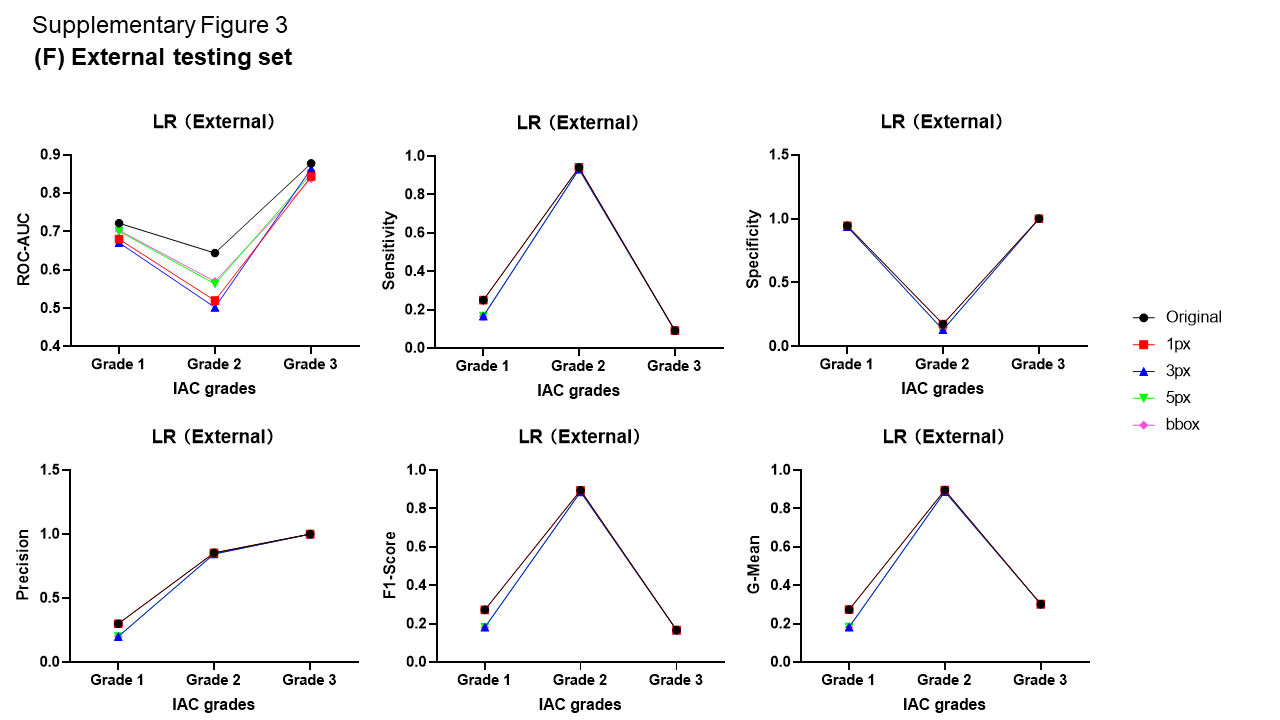


**Supplementary Figure 3. Comparison of model performance among different annotation strategies on histologic subtype classification and IAC grade stratification.** AUC, sensitivity, specificity, precision, F1-score, and G-mean of selected ML algorithm were calculated based on different annotation strategies for histologic subtype classification (MLP with 1-pixel annotation strategy) on the internal (A), independent (B), and external (C) testing sets, and for IAC grade stratification (LR with optimal annotation strategies) on the internal (D), independent (E), and external (F) testing sets.
